# Synchrotron Radiation Excited Fluorescence Micro-Analysis Using a New Imaging Technique

**DOI:** 10.6028/jres.093.085

**Published:** 1988-06-01

**Authors:** A. Knöchel, M. Bavdaz, N. Gurker, P. Ketelsen, W. Petersen, M. H. Salehi, T. Dietrich

**Affiliations:** Universität Hamburg, Institut für Anorganische und Angewandte Chemie, Martin-Luther-King-Platz 6, D-2000 Hamburg, FRG

## Introduction

The great importance and large field of applications the x-ray fluorescence analysis (XFA) found in the last decade are due to its extraordinary qualities. It is a very flexible method, it is nondestructive, fast and requires only a very simple sample preparation. It is not necessary to perform the analysis in vacuum, so samples containing volatile components pose no problem (e.g., biological and medical samples).

The penetration depth of x-rays ranges from a few µm up to several 100 µm and is thus much greater than that of electrons (used to excite the sample with scanning electron microscopy). The analyzed volume of the sample thus increases and results in a high sensitivity of the method. Additionally, x-rays cause much less radiation damage than corpuscular beams [1].

**Figure 1:**
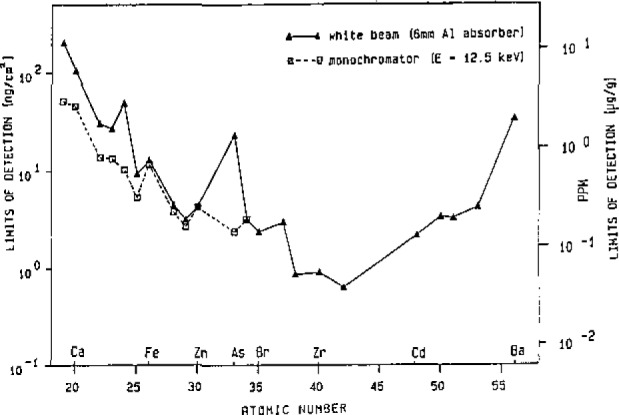
Detection limits for SYXFA [2].

Using synchrotron radiation as a very powerful primary x-ray source, the sensitivity of the method is very high and is comparably good for all elements having Z⩾ 18 [2,3]. Figure 1 shows the detection limits for synchrotron radiation excited x-ray fluorescence analysis (SYXFA). A 1 mg/cm^2^ multielement sample was measured for 300 s using a beam diameter of 0.5 mm. The solid line indicates the detection limits measured using a white beam, the dotted line those using a graphite monochromator adjusted to eliminate the As-Kα/Pb-Lα interference.

The main advantages of applying synchrotron light as the primary radiation source in XFA could be summarized as follows:

- The high photon flux makes filtering, monochromatizing and masking of the primary beam feasible.
- A very broad continuous white spectrum allows simultaneous multi-elemental analysis featuring very good limits of detection (absolute ⩾ 10^−13^ g, relative ⩾0.1 µg/g).
- Synchrotron radiation is linearly polarized in the storage-ring plane, thus improving the signal-to-noise ratio significantly.
- The source size is very small and the synchrotron beam is very well collimated, so a high spatial resolution is achievable (see later).
- Since the properties of synchrotron radiation are calculable, the evaluation of the measured data becomes easier and more reliable.

We are applying SYXFA to help solving different analytic problems that hardly could be approached with other methods. For example, we analyzed cosmic dust particles smaller than 10 µm in diameter to find out their composition. A second example is the analysis of the printing ink on very precious documents, including the 42-line bible by Gutenberg.

## X-Ray Imaging

SYXFA becomes even more interesting if one succeeds in combining spatial resolving analysis with the high sensitivity of the method.

X-ray imaging has experienced a renaissance in the last few years; microscopic details of specimens can now be imaged [4], but unfortunately only with soft x-rays. Zone plates show a low effective transmission (typically 10%) even in the soft x-ray region; for energies above 1 keV they are no longer applicable.

Reflecting optics also suffer from various difficulties; for use in microscopy, such optics are extremely complicated to make, since very high accuracies are required. The otherwise most promising multilayer reflection optics act as a relatively narrow band-pass filter and work only for a single defined x-ray energy.

**Figure 2:**
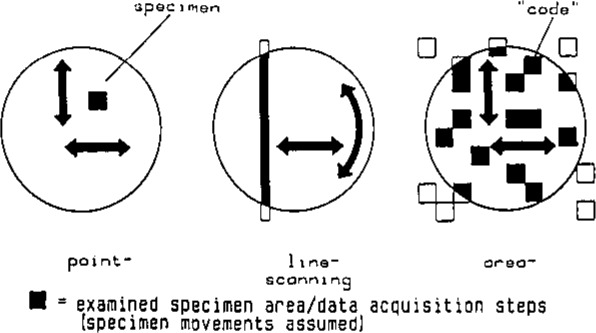
Different methods of x-ray scanning [7].

We are therefore going another way to reach the goal of achieving high resolution imaging and still maintaining a good signal-to-noise ratio; using coded imaging techniques we are increasing the interaction area of the incident beam with the sample (fig. 2) [5–7]. Line-scanning increases the interaction area by the linear dimension of the measured image, and area-scanning enables us to irradiate up to 50% of the sample.

**Figure 3:**
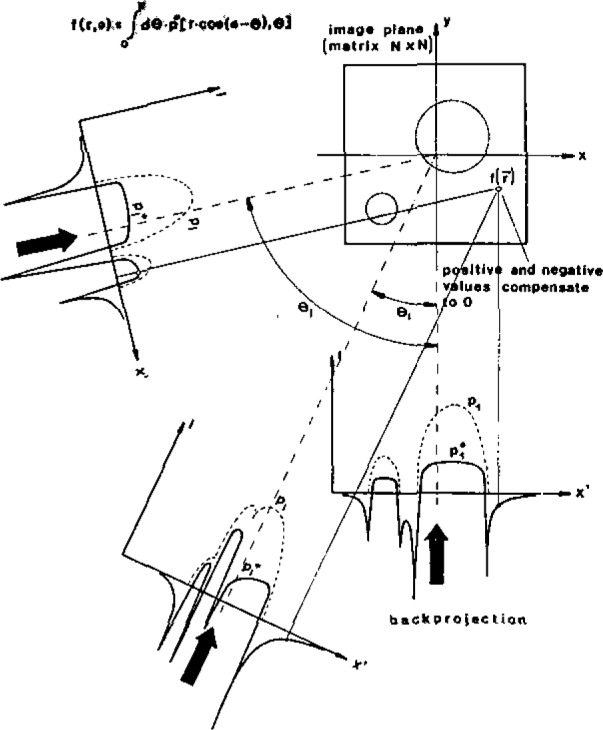
Filtered backprojection [8].

Line-scanning requires a linear and a rotational motion; the set of data-points measured at a given rotation angle is called a “profile.” Figure 3 shows schematically the process of back-projecting the measured data [8]; the profiles have to be filtered prior to their backprojection in order to avoid reconstruction errors [9]. Since each data-point contains information about several sample-pixels, the loss of some data-points has very little influence on the resulting image.

**Figure 4:**
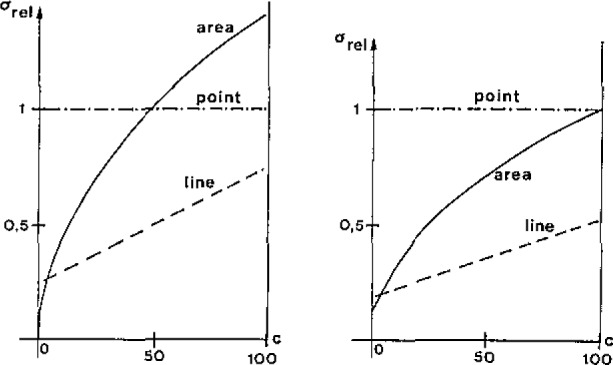
Estimated errors (σ_rel_) in reconstructed images versus signal area (*c*,%) for different scanning methods (for the right plot the presence of a constant background was assumed).

An estimation of the relative errors in the reconstructed image is given in figure 4 (the signal area is defined as the percentage of the sample area contributing to the signal; 0/1-contrast was assumed).

**Figure 5:**
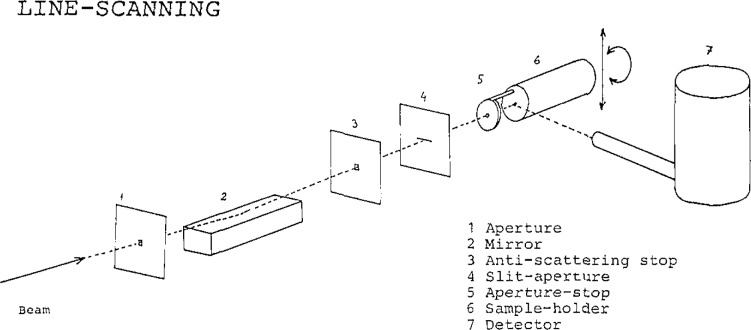
Experimental setup.

The experimental set-up is outlined in figure 5. Several stops reduce the scattered radiation; the total-reflecting mirror is used to cut off x-rays that are too hard. The slit-aperture (No. 4 in fig. 5) is made very accurately and has a width down to 2 µm. The alignment of the rotation axis of the sample has to be adjusted very carefully with respect to the synchrotron-beam axis. Both axes have to be parallel better than a few tenths of an arc minute.

**Figure 6:**
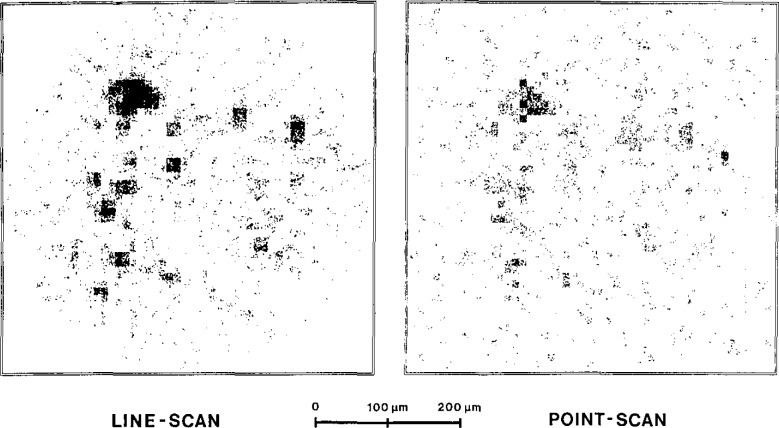
Comparison: line- and point-scanning (experimental; Sn grains in organic polymer matrix).

Figure 6 compares an image measured by linescanning to another measured by conventional point-scanning. In both cases the sample was measured under the same conditions (geometry, measuring time, filtering, parameters of electronics and accelerator).

## Discussion

By applying the line-scanning technique to scan the sample, it was possible to reach spatial resolutions down to 3 µm with SYXFA, still being able to measure trace elements. Nevertheless, this is a very demanding technique. A synchrotron radiation laboratory is necessary, a computer is needed to decode (backproject) the measured data, and a very tricky alignment of the experiment has to be performed.

Considering the fascinating properties of this SYXFA-microprobe, many interesting fields of applications promise interesting results, e.g., aerosols, pigments of printing-ink on historic documents or oil paints on canvas, blood particles, diffusion processes and structured multilayers (e.g., electronic circuits).

The experiments were generously supported by the “Bundesministerium für Forschung und Technologic.” Thanks are due to the HASYLAB-team for their professional assistance.
